# The Intriguing Evolutionary Journey of Enteroinvasive *E. coli* (EIEC) toward Pathogenicity

**DOI:** 10.3389/fmicb.2017.02390

**Published:** 2017-12-05

**Authors:** Martina Pasqua, Valeria Michelacci, Maria Letizia Di Martino, Rosangela Tozzoli, Milena Grossi, Bianca Colonna, Stefano Morabito, Gianni Prosseda

**Affiliations:** ^1^Istituto Pasteur Italia, Department of Biology and Biotechnology “C. Darwin”, Sapienza Università di Roma, Rome, Italy; ^2^European Union Reference Laboratory for Escherichia coli, Department of Veterinary Public Health and Food Safety, Istituto Superiore di Sanità, Rome, Italy

**Keywords:** pathogenic *E. coli*, enteroinvasive *E. coli* (EIEC), *Shigella*, bacterial evolution, emerging EIEC

## Abstract

Among the intestinal pathogenic *Escherichia coli*, enteroinvasive *E. coli* (EIEC) are a group of intracellular pathogens able to enter epithelial cells of colon, multiplicate within them, and move between adjacent cells with a mechanism similar to *Shigella*, the ethiological agent of bacillary dysentery. Despite EIEC belong to the same pathotype of *Shigella*, they neither have the full set of traits that define *Shigella* nor have undergone the extensive gene decay observed in *Shigella*. Molecular analysis confirms that EIEC are widely distributed among *E. coli* phylogenetic groups and correspond to bioserotypes found in many *E. coli* serogroups. Like *Shigella*, also in EIEC the critical event toward a pathogenic life-style consisted in the acquisition by horizontal gene transfer of a large F-type plasmid (pINV) containing the genes required for invasion, intracellular survival, and spreading through the intestinal mucosa. In *Shigella*, the ample gain in virulence determinants has been counteracted by a substantial loss of functions that, although important for the survival in the environment, are redundant or deleterious for the life inside the host. The pathoadaptation process that has led *Shigella* to modify its metabolic profile and increase its pathogenic potential is still in infancy in EIEC, although maintenance of some features typical of *E. coli* might favor their emerging relevance as intestinal pathogens worldwide, as documented by recent outbreaks in industrialized countries. In this review, we will discuss the evolution of EIEC toward *Shigella*-like invasive forms going through the epidemiology, including the emergence of new virulent strains, their genome organization, and the complex interactions they establish with the host.

## Introduction

*Escherichia coli* is not only a harmless commensal of the human and animal intestine but also a major cause of morbidity and mortality (Kaper et al., 2004; Wirth et al., 2006). Indeed, many pathogenic *E. coli* have been described as cause of diseases both in healthy and immunocompromised individuals. Based on the specific virulence factors and pathogenicity processes, pathogenic *E. coli* have been subdivided into different pathogroups, that can be broadly grouped as DEC (or intestinal) or extraintestinal *E. coli* (ExPEC) (Kaper et al., 2004; Croxen and Finlay, 2009; Gomes et al., 2016). DEC include at least six major pathotypes differing in virulence mechanisms, infectious processes, and damages provoked to the target cells: enteropathogenic *E. coli* (EPEC), Shiga toxin-producing *E. coli* (STEC), enterotoxigenic *E. coli* (ETEC), enteroinvasive *E. coli* (EIEC), enteroaggregative *E. coli* (EAEC), diffusely adherent *E. coli* (DAEC), as well as adherent invasive *E. coli* (AIEC), a recently identified pathotype. As for ExPEC, the most common strains belong to two different pathotypes targeting different body compartments: uropathogenic *E. coli* (UTI) and neonatal meningitis *E. coli* (NMEC).

The presence of so many different pathotypes exemplifies the remarkable plasticity of *E. coli* genome, which is characterized by an extremely large pangenome of approximately 20,000 genes in contrast to a common core of about 1700 genes (Rasko et al., 2008; Touchon et al., 2009). Those that vary among different pathogenic *E. coli* strains have been acquired by intense HGT and are often conveyed by mobile genetic elements (Touchon et al., 2009; Dobrindt et al., 2010; van Elsas et al., 2011).

Among the DEC pathotypes, EIEC are etiological agents of bacillary dysentery in humans, particularly in low-income countries (Croxen et al., 2013; Gomes et al., 2016). The pathogenesis of EIEC infection is characterized by the ability of bacteria to invade the human colonic mucosa, conferred by the expression of chromosomal and plasmid-borne genes (Harris et al., 1982; Sansonetti et al., 1982; Hale et al., 1983; Kaper et al., 2004). Following penetration into colonic epithelial cells, EIEC replicate intracellularly and spread to adjacent cells causing the inflammatory destruction of the intestinal epithelial barrier. This provokes the characteristic dysentery syndrome, usually self-limiting, characterized by the presence of blood, mucus, and leukocytes in stools (DuPont et al., 1971; O’Brien et al., 1979; Taylor et al., 1988). The clinical illness caused by EIEC is similar to that induced by *Shigella* spp. (Formal and Hornick, 1978; Small and Falkow, 1988), with whom they are closely related in their virulence and other phenotypic properties (Kopecko et al., 1985; Lan et al., 2004). Notwithstanding the similarities in the invasion mechanisms, the infectious dose of EIEC has been observed to be much higher than that of *Shigella* and the diseases caused by EIEC appear in some cases to be milder (DuPont et al., 1971).

Despite several studies, whether EIEC are precursors of the “full blown” pathogen *Shigella*, or not is still under debate. In this review, we will attempt at tracing the evolutionary pathway of EIEC considering their epidemiology, the complex mechanisms of their interaction with host cells, the key steps that could have characterized their evolution from a commensal life style toward pathogenicity, and the organization of their genome, including the description of the major traits of emerging EIEC clones.

## Epidemiology of Enteroinvasive *E. coli* (EIEC)

The first report of an EIEC strain dates back at 1947 (Ewing and Gravatti, 1947). At that time, it was defined as “paracolon bacillus” but the strain was later identified as an O124 *E. coli*. In the 1950s and 1960s, other *E. coli* strains, isolated from dysentery and initially classified as *Shigella manolovi*, *S. sofia*, *Shigella* strain 13, and *S. metadysenteriae*, due to their ability to cause experimental keratoconjunctivitis in guinea pigs, were later renamed as EIEC (Manolov, 1959; Rowe et al., 1977; Edwards and Ewing, 1986). Their biochemical characters were first described in 1967 (Sakazaki et al., 1967; Trabulsi et al., 1967).

Enteroinvasive *E. coli* and *Shigella* spp. share several phenotypic and genotypic characteristics, often making the discrimination between the two genera challenging (Silva et al., 1980; Toledo and Trabulsi, 1983; Bando et al., 1998; Lan and Reeves, 2002; Pavlovic et al., 2011; van den Beld and Reubsaet, 2012), especially in case of shared serogroups. This difficulty biases the interpretation of the epidemiological information available, hindering the evaluation of the real burden of EIEC infections. As a matter of fact, both EIEC and *Shigella* spend much of their life cycle within the eukaryotic cells, possessing the ability to use nutrients coming from the host environment. Similarly to *Shigella*, most EIEC strains are unable to decarboxylate lysine, lack the ability to ferment lactose, and are generally non-motile, with the exception of strains belonging to a few serogroups (Silva et al., 1980; Farmer et al., 1985; Bando et al., 1998; Casalino et al., 2003; Tozzoli and Scheutz, 2014).

A limited set of serotypes have been assigned to EIEC, namely O28ac:H-, O29:H-, O112ac:H-, O115:H-, O121:H-, O124:H-, O124:H7, O124:H30, O124:H32, O135:H-, O136:H-, O143:H-, O144:H-, O144:H25,O152:H-, O159:H-, O159:H2, O164:H-, O167:H-, O167:H4, O167:H5, O173:H-, and recently O96:H19 (Voeroes et al., 1964; Silva et al., 1980; Gomes et al., 1987, 2016; Orskov et al., 1991; Matsushita et al., 1993; Escher et al., 2014; Tozzoli and Scheutz, 2014; Michelacci et al., 2016; Newitt et al., 2016). Some of these EIEC-associated O antigens, such as O28, O112ac, O121, O124, O143, O144, O152, and O167, are identical to O antigens present in *Shigella* spp. (Cheasty and Rowe, 1983; Tozzoli and Scheutz, 2014).

Enteroinvasive *E. coli*-infected humans seem to be the major source of infection, as no animal reservoirs have been identified, and transmission uses mainly the oral–fecal route. Although EIEC infections occur worldwide, these are particularly common in low-income countries where poor general hygiene favors their spreading (Chatterjee and Sanyal, 1984; Beutin et al., 1997; Kaper et al., 2004; Vieira et al., 2007).

Enteroinvasive *E. coli* incidence has been estimated in several countries, and it differs depending on the region (Gomes et al., 2016). Discrepancies among some of the reports can be observed, probably due to the difficulty in discriminating between *Shigella* and EIEC. In certain countries of Latin America and Asia, namely Chile, Thailand, India, and Brazil, EIEC were found to be common diarrheagenic pathogens (Chatterjee and Sanyal, 1984; Faundez et al., 1988; Echeverria et al., 1992; Blake et al., 1993; Levine et al., 1993), with frequent reports of asymptomatic infected subjects excreting the pathogen (Beutin et al., 1997). In industrialized countries, EIEC infections have been mainly described as travel-related, being reported in returning travelers from high-incidence countries (Wanger et al., 1988; Beutin et al., 1997; Svenungsson et al., 2000). Occasionally, food and water sources have been identified as vehicles of infection, but usually as a secondary contamination by a human source (Tozzoli and Scheutz, 2014).

Enteroinvasive *E. coli* cause sporadic cases of infection but have been implicated in outbreaks as well, sometimes involving large numbers of cases. In the 1970s a huge outbreak, affecting 387 patients and linked to cheese contaminated with an O124 *E. coli* strain, occurred in United States (Marier et al., 1973). Recently, an increase of cases of infections linked to an emerging EIEC clone has been observed in Europe, where in 2012 a large and severe outbreak of bloody diarrhea in Italy involving more than 100 individuals was reported (Escher et al., 2014; Pettengill et al., 2015). An EIEC O96:H19 strain, a serotype never described before for EIEC, was isolated and the suspected source of infection was traced to cooked vegetables (Escher et al., 2014). During the outbreak investigation an EIEC O96:H19 strain was also isolated from two asymptomatic food handlers working in the canteen linked with the outbreak, supporting the hypothesis of a secondary contamination of the vegetables during post-cooking handling procedures (Escher et al., 2014). In 2014, two linked outbreaks of gastrointestinal disease occurred in the United Kingdom, involving more than 100 cases of infection. One of the episodes was associated to the consumption of contaminated salad vegetables and, again, an O96:H19 EIEC was isolated from some of the patients and from vegetable samples (Newitt et al., 2016). Finally, an EIEC belonging to the same serotype was isolated in a case of traveler’s diarrhea in Spain in 2013 (Michelacci et al., 2016). Pheno-genotypic characterization of the strains involved in the three episodes suggests that the EIEC O96:H19 could be emerged as a result of the recent acquisition of the invasion plasmid by an *E. coli* strain (Michelacci et al., 2016).

## The Invasive Process

Similarly to *Shigella*, EIEC are responsible of bacillary dysentery (Taylor et al., 1988). However, the disease caused by EIEC is usually less severe than that induced by *Shigella* (DuPont et al., 1971). Following the discovery that EIEC strains carry a pINV plasmid identical to that of *Shigella* (Harris et al., 1982; Sansonetti et al., 1982; Hale et al., 1983) and that they can display a *Shigella*-like invasive behavior (Hale et al., 1985; Small and Falkow, 1988; Taylor et al., 1988), *in vitro* and *in vivo* studies have been extensively focused on *Shigella*, providing in-depth knowledge about its pathogenicity/virulence mechanisms. In recent years, the pathogenicity of EIEC has gained new interest and comparative analyses between EIEC and *Shigella* have been performed, aimed at understanding the different clinical outcome severity of the two infections (Moreno et al., 2009; Bando et al., 2010; Sanchez-Villamil et al., 2016). Here we first present the invasive process as it has been inferred from studies on *S. flexneri*. Then, we address what it is known about the difference between these two enteroinvasive bacteria.

In order to gain access to intestinal epithelia, bacteria first transit from the lumen to the submucosa by preferentially entering M cells in Peyer’s patches. After endocytosis by M cells bacteria are transcytosed toward the M cell pocket, where they meet, and are phagocytosed by resident macrophages. *Shigella* infection of macrophages is accompanied by the release of T3SS effectors and components that are recognized as PAMPs by NLRs, ultimately leading to pyroptosis with the release of proinflammatory cytokines, IL-1β and IL-18 (Ashida et al., 2011). The induction of macrophage cell death is pivotal for bacteria to invade enterocytes, though pyroptosis is a form of cell death that induces a massive inflammatory response. Once released from dying macrophages, invasive bacteria infect the neighboring enterocytes by entering through the basolateral surface. Here they are enclosed into a vacuole that is rapidly disrupted freeing them into the cytosol. Subsequently, the bacteria multiply and, using actin-based motility, spread to adjacent cells (Schroeder and Hilbi, 2008).

Inside epithelial cells, bacterial PAMPs and DAMPs are detected by various PRRs, including TLRs and NLRs, which stimulate host defense signal pathways such as those involving MAPKs and NF-κB leading to the secretion of proinflammatory cytokines (e.g., IL-8 and TNF-α) (Takeuchi and Akira, 2010). These molecules induce the recruitment of phagocytic cells to the infection site, initially facilitating the invasion process and eventually clearing the bacterial pathogens. In order to maximize invasion and permanence and save the replicative niche in epithelial cells, invading *Shigella* modulate host cell responses throughout the infection process by secreted effectors (Killackey et al., 2016). Induction of a very early inflammatory response upon invasion of epithelial cells is functional to bacterial spreading as it results in recruitment of polymorphonuclear leucocytes (PMNL), which migrate across the epithelium destabilizing the intercellular junctions and increasing the surface available for bacterial entry into target cells (Ashida et al., 2011). Several T3SS effectors, such as OspB, OspC1, and OspZ (Zurawski et al., 2009; Ambrosi et al., 2015; Mattock and Blocker, 2017), contribute to promote inflammation at early stages of the infection process. They mainly act by enhancing activation of MAPK and NF-κB pathways, which are involved in the control of the production of PMNL chemoattractants, including IL-8, whose secretion triggers PMNL migration in a basolateral to apical direction causing epithelial barrier disruption. However, though this early inflammatory response is essential to initiate infection, it would also contribute toward rapidly clearing the infecting agents. Thus, to establish infection, at later stages *Shigella* must overcome the host innate response. This is achieved by delivering T3SS effectors, whose function is aimed mainly at inhibiting MAPK and NF-κB signaling pathways with the consequent decrease of inflammatory chemokine and cytokine production (Killackey et al., 2016; Mattock and Blocker, 2017).

An important obstacle *Shigella* must tackle during the invasion of the epithelial tissue is host cell targeting and degradation by autophagy. Several studies have demonstrated that *Shigella* are particularly exposed to autophagy targeting only when they are associated to cell membranes. Two bacterial factors, IcsB and VirA, have been implicated in bacterial evasion of autophagy targeting by interfering with LC3 recruitment and by allowing bacteria to escape from LC3-positive vacuoles (Ogawa et al., 2005; Baxt and Goldberg, 2014; Campbell-Valois et al., 2015).

Typically, intracellular pathogens need to save their host to establish a successful infection. As part of their pathogenic mechanism *Shigella* employ several countermeasures to avoid premature cell death to maintain their epithelial replicative niche. The early stage of infection is characterized by induction of DNA damage and genotoxic stress, which lead to activation of p53 and stimulation of apoptosis. Apoptotic cell death is prevented by the activity of the T3SS effectors VirA and IpgD, which promote p53 degradation and activate the PI3K/Akt pro-survival pathway, respectively, and by the pilus component protein FimA, which inhibits cytochrome c release by mitochondria (Mattock and Blocker, 2017).

As discussed above, EIEC share many aspects of the *Shigella* infection process that involves crossing of intestinal epithelial barrier, killing of resident macrophage cells, invasion of enterocytes, intra-cellular replication, and dissemination from cell to cell without extracellular steps (Croxen and Finlay, 2009). Moreover, EIEC express the same virulence factors found in *Shigella* (Parsot, 2005). However, the infectious dose required for EIEC to cause disease is higher than that of *Shigella* and the disease caused by EIEC appears to be milder (DuPont et al., 1971), suggesting differences between EIEC and *Shigella* in sensing and shaping the host environment, which, in turn, would influence the pathways toward virulence. To date only few studies have investigated the differences in the infectiveness between EIEC and *Shigella*. Moreno et al. (2009) detailed for the first time the relationship between the expression of some genes crucial for the infection process and the reduced ability of EIEC to cause disease. This is well supported by their Serény tests in guinea pigs, showing how the signs of keratoconjunctivitis induced by *Shigella* appear earlier and are more severe as compared to those caused by EIEC. Using an epithelial cell model, the authors also demonstrate that, although *Shigella* and EIEC display similar invasion ability, EIEC disseminates less efficiently, producing smaller plaques in plaque assays. As compared to *Shigella* the overall behavior of EIEC apparently reflects a reduced expression of key virulence genes, during both invasion and cell-to-cell spreading, except for *virF* that is expressed at higher levels by intracellular EIEC than *Shigella* during the dissemination step. This apparent discrepancy may be explained in the light of recent results showing that *Shigella virF* is transcribed into two mRNAs, with the shortest one encoding a smaller protein that negatively regulates transcription of full-length mRNA and, consequently, the expression of the VirF regulator (Di Martino et al., 2016b). Since in the real-time PCR experiments carried out by Moreno et al. (2009) *virF* expression was assayed by using primers that did not discriminate between the two mRNAs, comparative *virF* expression studies between *Shigella* and EIEC deserve further investigations to deeper analyze potential differences.

A more recent work has compared the host cell response to infection by different *E. coli* pathotypes, including EIEC, and by *Shigella*. The kinetic of NF-κB and ERK1/2 activation in HT-29 epithelial cells shows only a slightly higher p65 phosphorylation after 4 h of infection with *Shigella* as compared with EIEC. Conversely, although following a similar kinetics, the accumulation of phosphorylated ERK1/2 is much higher in cells infected with EIEC at 4 h post-infection. Despite these differences, HT-29 cells infected with EIEC or *Shigella* release comparable amounts of cytokines, as IL-8 and TNF-α with similar kinetics (Sanchez-Villamil et al., 2016). The phosphorylation of ERK1/2 and p38 is controlled by the phosphothreonine lyase activity of OspF, to which both anti-inflammatory (Arbibe et al., 2007) and pro-inflammatory roles (Reiterer et al., 2011) have been attributed. Since both *Shigella* and EIEC express OspF, it is reasonable that additional factors are involved in determining the different ERK1/2 phosphorylation profile and the outcome of MAPK activation.

The key step in invasion of the epithelial cells resides in the ability of EIEC and *Shigella* to escape from macrophages after phagocytosis by induction of caspase 1-dependent cell death. It has been reported that, as compared to *Shigella*, EIEC have a decreased capacity to escape from murine J774 macrophages and are less efficient in cell killing during the first 4 h of infection (Bando et al., 2010). This likely depends on differences in the expression of some virulence genes. In particular, as compared to *Shigella* the expression of the *ipaC* gene is reduced in intracellular EIEC at all the time points after infection. As for the release of pro- and anti-inflammatory cytokines (as TNF-α, IL-1, and IL-10) by infected cells, contrasting results exist. While no significant differences between EIEC and *Shigella*-infected J774 cells (Bando et al., 2010) have been reported, other studies carried out using human THP-1 cells differentiated into macrophages (Sanchez-Villamil et al., 2016) have shown that *Shigella* infection results in higher secretion of both pro-inflammatory and anti-inflammatory cytokines.

To date, banking on the modest amount of data available from *in vitro* infection of macrophage-like cells and epithelial cells, the milder disease caused by EIEC appears to be mainly associated to a lower expression of key virulence genes involved in phagosomal escape inside host cells and in dissemination among epithelial cells (Moreno et al., 2009; Bando et al., 2010). There are no obvious differences in the inflammatory response by epithelial cells, at least as far as the secretion of IL-8 and TNF-α is concerned, neither at early nor at late times of infection. Despite this cytokine profile, the activation state of ERK1/2 MAPK seems to be more elevated in epithelial cells infected with EIEC than in those infected with *Shigella* (Sanchez-Villamil et al., 2016). Deeper investigations will clarify to what extent this may depend on differences in manipulating certain cell signaling pathways and on differences in the activity of bacterial factors involved therein.

## The Major Virulence Trait of EIEC: The Large Virulence Plasmid pINV

The evolution of *E. coli* toward pathogenic phenotypes has been determined, as in many other bacterial pathogens, mainly by two mechanisms: the acquisition of virulence genes by HGT as parts of plasmids, phages, transposons, or PAI and the loss or modification of genes of the core genome. While the first mechanism plays a crucial role in the colonization of a new host environment, the latter, known as pathoadaptation, strongly contributes to drive the evolution of bacteria toward a more pathogenic phenotype (Kaper et al., 2004; Dobrindt et al., 2010).

**FIGURE 1:**
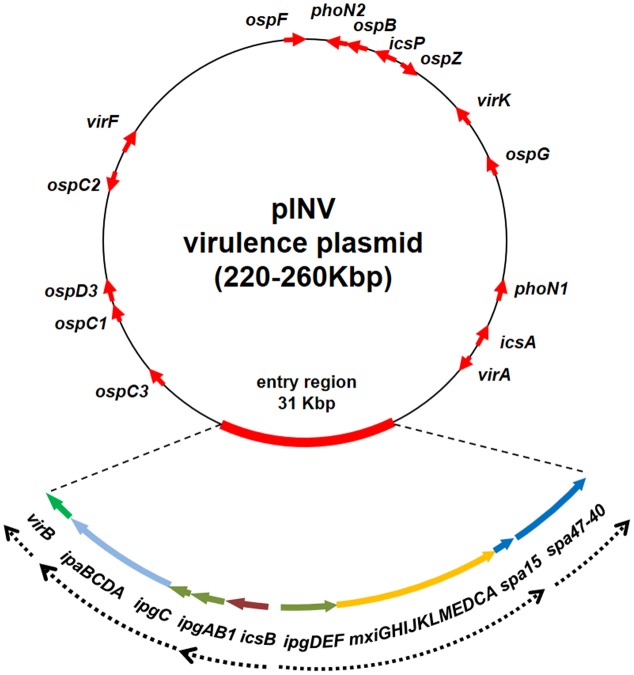
Genetic map of the pINV of *Shigella* and EIEC strains. The red arrows indicate major virulence determinants. Due to the variability in position and number, the *ipaH* genes are not shown. The genetic organization of the entry region is shown in detail, with dashed arrow lines indicating known transcriptional units. The entry region organization is based on the sequence of plasmid pWR100 (Venkatesan et al., 2001) while the entire plasmid is freely drawn to provide the layout of a typical pINV plasmid (the figure is not to scale).

It is widely acknowledged that, as in *Shigella*, in EIEC the critical event in the transition toward a pathogenic lifestyle has been the acquisition of a large F-type plasmid (pINV) which encodes the molecular machinery required for invasion, survival, and diffusion of the bacterium within the host (Harris et al., 1982; Sansonetti et al., 1982; Hale et al., 1983; Small and Falkow, 1988; **Figure 1**). The pINV plasmid has been found only in the *Shigella*/EIEC pathotype and its loss is a very rare event, which determines an avirulent phenotype.

The genetic organization of the pINV is very complex (Johnson and Nolan, 2009). As a matter of fact these plasmids are made up of a mosaic of genes of various origins and harbor traces of four different plasmids (Buchrieser et al., 2000; Venkatesan et al., 2001; Escobar-Páramo et al., 2003). pINV isolated from EIEC share wide regions of high structural and functional homology and are interchangeable with those isolated from *Shigella* strains (Hale et al., 1983; Lan et al., 2001; Johnson and Nolan, 2009). pINV share with IncFIIA plasmids high homology in the regions involved in replication (*rep*) and conjugation (*tra*) (Makino et al., 1988) and stable inheritance of pINV is ensured by the presence of several plasmid segregation and maintenance systems (Lan et al., 2001). Due to large deletions in the *tra* region, pINV are not capable of self-transfer by conjugation, but they can be mobilized by other conjugative plasmids. All over the plasmid genome, an astonishing number of ISs is present as a mixture of complete and incomplete IS elements repeated several times, confirming the relevant role played by ISs in pINV assembly and evolution (Buchrieser et al., 2000; Venkatesan et al., 2001). Most ISs are related to known elements while others represent novel ISs. Among the latter, ISEc11, an IS belonging to the IS1111 family, is widespread and functional in pINV from EIEC while only defective copies are present in the *Shigella* pINV plasmids (Prosseda et al., 2006).

In the pINV there is only one large (31 kb) region, which does not host any IS elements. This is the so-called entry region, which displays a PAI-like structure (Buchrieser et al., 2000; Venkatesan et al., 2001). It is composed by two large, divergently transcribed gene clusters coding for a T3SS apparatus (Mxi and Spa), for most of its effectors (IpaB, IpaC, and IpaD) with their chaperons (IpgA, IpgC, IpgE, and Spa15), and for two transcriptional regulators (VirB and MxiE), both required for the activation of most virulence genes (Schroeder and Hilbi, 2008; **Figure 1**). The entry region is extremely conserved among *Shigella* and EIEC pINV plasmids (Lan et al., 2001). Albeit it had been initially proposed as a PAI, likely acquired in a single recombination event, it lacks the presence of flanking tRNA sequences and at least remnants of a recombinase-encoding gene. It is therefore unclear if the acquisition of the entry region has occurred independently from its insertion into tRNA sequences or if the absence of tRNA genes may have resulted from rearrangement following gene transfer. The latter hypothesis is supported by the fact that the entry region is flanked by truncated IS elements, suggesting that rearrangements may have occurred after its acquisition *en bloc* by the plasmid (Buchrieser et al., 2000). The T3SS encoded by the entry region plays a critical role in the bacterial invasive process, since it delivers a large number of effectors involved in the reorganization of the host cell actin cytoskeleton and in the modulation of cell signaling pathways to evade the host immune response (Mattock and Blocker, 2017). With the exception of few proteins of the IpaH family, which are chromosomally encoded, all T3SS effectors are encoded by pINV genes located within or outside the entry region. Since the entry region is highly conserved, the phylogenetic analysis of three of its genes (*ipgD*, *mxiC*, and *mxiA*) has allowed differentiating pINV from *Shigella* spp. and EIEC into two forms, A and B, with the first one predominantly associated with EIEC strains (Lan et al., 2001, 2004).

Besides the large PAI-like region, a small islet carries the genes coding for IcsA (a protein responsible for the bacterial motility inside the cytoplasm), VirA (a GTPase-activating protein), and RnaG (a regulatory sRNA negatively controlling *icsA* expression) (Giangrossi et al., 2010; Tran et al., 2011; Dong et al., 2012). Other genes encoding proteins crucial for the invasive process cover the pINV plasmid including the OspG and OspF proteins which interfere with the host innate immune response (Kim et al., 2005; Arbibe et al., 2007), the PhoN2 protein required for IcsA localization (Scribano et al., 2014), and the IpaH proteins which interfere with the host protein degradation (Ashida and Sasakawa, 2015; **Figure 1**). Moreover, in contrast to the other two virulence regulatory genes (*virB* and *mxiE*), the *virF* gene, coding for the primary virulence regulator, is located on a “desert island” surrounded by several IS sequences and far away from all other virulence genes, including those under its direct control, *virB* and *icsA* (Di Martino et al., 2016a). While the CG content of *virF* is only slightly lower as compared to that of the entry region (Buchrieser et al., 2000), its position suggests that it has been acquired independently to promote the expression of the virulence genes. VirF is also involved in the activation of some chromosomal genes, indicating that it acts as global regulator and that its acquisition by HGT has contributed to a reshaping of the core genome, easing the adaptation of bacteria to the host environment (Barbagallo et al., 2011; Leuzzi et al., 2015).

The mechanisms involved in the activation of the pINV virulence genes have been extensively studied both in EIEC and in *Shigella* (Dagberg and Uhlin, 1992; Prosseda et al., 2002). They rely on a sophisticated regulatory cascade involving global and specific regulators, encoded by both, pINV and the chromosome. Outside the human host, the nucleoid-associated protein H-NS represses each of the key promoters of the pINV virulence genes (Dorman, 2004). In response to environmental conditions found in the human intestine, the transcriptional activation of the invasive operons is triggered by an increased level of VirF counteracting H-NS repression at the *icsA* and *virB* promoters (Prosseda et al., 2004). Then VirB activates most operons within the entry region, including the gene for the last regulator (*mxiE*), as well as all other virulence genes scattered along the pINV genome, except *icsA*. Finally, MxiE, assisted by IpgC, activates the transcription of genes encoding the late effectors (Schroeder and Hilbi, 2008).

As in other pathogenic *E. coli*, also in EIEC the virulence genes are stably maintained on an extrachromosomal element (Johnson and Nolan, 2009). Nevertheless, it has been reported that the pINV of EIEC strain HN280 is able to integrate into the host chromosome and that integration results in silencing of all pINV-encoded virulence genes also under host temperature conditions (Zagaglia et al., 1991). Silencing was shown to depend on a severe reduction of *virB* transcription, likely dependent on the inability of VirF to counteract the negative control of H-NS at the *virB* promoter when it is chromosomally located (Colonna et al., 1995). This has led to the hypothesis that the presence of virulence genes on the pINV is the result of an evolutionary pathway toward the optimization of gene expression.

## Evolution of EIEC

**FIGURE 2:**
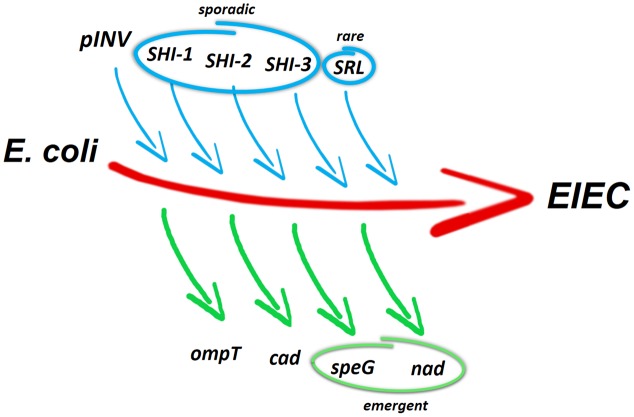
Genetic events contributing to the evolution of EIEC from ancestral commensal *E. coli*. The acquisition of the pINV by HGT is a major evolutionary event toward pathogenicity. This can be accompanied by the sporadic acquisition of entire or incomplete SHI-1 PAI and incomplete SHI-2 and SHI-3 PAIs. Rarely, also incomplete SRL PAI are acquired by EIEC genomes. The absence of *ompT* and the loss of cadaverine synthesis (usually resulting from *cadC* silencing) counterbalance the gain of virulence-associated determinants. The inactivation of *speG* (involved in spermidine acetylation) and *nad* (involved in NAD biosynthesis) is regarded as emergent pathoadaptive mutations in EIEC.

The studies on the evolutionary origin of the *Shigella*/EIEC pathovar have led to two major hypotheses. The pINV could have been acquired only once by an ancestral *E. coli* that subsequently gave rise to the different *Shigella*/EIEC lineages (Escobar-Páramo et al., 2003; Zuo et al., 2013), as suggested by the inability of the plasmid to autonomously undergo horizontal transmission. Alternatively, the different *Shigella*/EIEC strains could have arisen from different *E. coli* that had acquired the pINV independently, e.g., from an unknown donor or from other *Shigella*/EIEC that already harbored it. This view is supported by the diversity of the genotypes within the *Shigella*/EIEC pathovar, revealed by phylogenetic analyses of chromosomal genes and by genome comparison (Pupo et al., 2000; Hazen et al., 2016; Pettengill et al., 2016). Besides the large pINV, several virulence genes have been acquired on the chromosome of *Shigella* and EIEC as part of PAIs (**Figure 2**). The PAIs described so far for *Shigella* (SHI islands) carry genes encoding different traits, including an enterotoxin and a cytotoxic protease (SHI-1) and systems involved in iron uptake and evasion of immune response (SHI-2 and SHI-3 in *S. boydii*), in the modification of O antigens (SHI-O) or in multi-drug resistance (SRL) (Schroeder and Hilbi, 2008). Recently, 20 genomes from EIEC belonging to different serotypes have been compared with those of reference strains belonging to diverse *E. coli* pathovars and *Shigella* species. This comparison highlights the existence of at least three distinct lineages containing only EIEC strains and suggests a convergent evolution of non-pathogenic *E. coli* toward invasive phenotype (Hazen et al., 2016). An *in silico* search for protein-encoding genes of SHI-1, SHI-2, SHI-3, SHI-O, and SRL indicates that, with the exception of SHI-O, portions of the other PAIs are present in EIEC genomes in a lineage-specific manner (Hazen et al., 2016). Interestingly, while a whole SHI-1 Island has never been detected in EIEC, SHI-1 fragments of different length have been found in all EIEC genomes. However, the ShET1 toxin genes, typically harbored by SHI-1 in *S. flexneri* genomes, were found only in EIEC strains of lineage 2. In the case of virulence genes associated with SHI-2, the *shi*A gene, involved in the reduction of the host inflammatory response, is absent in all EIEC lineages, while *shiD*, which provides immunity to colicins, is present in all EIEC of lineages 1 and 2. An entire SHI-3 PAI, typically associated with *S. boydii* strains, has been detected only in few EIEC strains of lineages 1 and 2, while portions of it, including the genes encoding for aerobactin-mediated iron uptake, are found in all three lineages. As for the large SRL PAI, widely distributed among *Shigella* spp. and containing a cluster of multiple antibiotic resistance determinants (Turner et al., 2003), only a few of its genes are present in EIEC genomes.

The variable presence of the PAIs in EIEC confirms the phylogenetic diversity among EIEC and *Shigella* and further supports the hypothesis that the EIEC pathovar has not a single origin but rather stems from multiple lineages (Hazen et al., 2016; Michelacci et al., 2016; Pettengill et al., 2016).

A significant complementary step toward the pathogenic lifestyle has been pathoadaptation, the inactivation, or loss of several chromosomal genes, which negatively interfere with the expression of virulence factors required for survival within the host. The antivirulence loci identified encode a broad spectrum of functions, confirming that adaptation to the new host environments is the result of long and ordered process targeting core genome determinants (Casalino et al., 2003; Di Martino et al., 2013b; Campilongo et al., 2014).

Despite the close similarity of the *Shigella* and EIEC pathogenicity process, it is well known that EIEC have a metabolic activity more similar to *E. coli* and have not undergone the intense gene decay observed in *Shigella* (Silva et al., 1980; Pettengill et al., 2016). It is therefore not surprising that the pathoadaptation in EIEC has not reached a level comparable to *Shigella* (Prosseda et al., 2012) and that most of the antivirulence loci characterized in *Shigella* are still encoding functional products in EIEC. One of the pathoadaptive mutations conserved both in EIEC and in *Shigella* is the deletion of the *ompT* gene, located within the defective lambdoid prophage DLP12 (Nakata et al., 1993; **Figure 2**). The OmpT protease triggers the degradation of IcsA protein and therefore negatively interferes with host cell invasion by drastically reducing the ability of *Shigella* to spread into adjacent epithelial cells. Considering that the loss of OmpT is widespread, it is as yet unclear if *E. coli* lineages that gave rise to the *Shigella*/EIEC pathovar have not hosted DLP12 *ab initio* or if the prophage has been excised during the pathoadaption process (Bliven and Maurelli, 2012; Leuzzi et al., 2017).

Another typical pathoadaptive mutation of *Shigella* spp. is the inability to catabolise lysine, due to the silencing of lysine decarboxylase (LDC) activity (Prosseda et al., 2007). The LDC^-^ phenotype, which is found also in most EIEC, is determined by mutations in the *cad* locus, which hamper the synthesis of cadaverine. Cadaverine is a polyamine that interferes with pathogenicity by blocking the release of *Shigella* into the cytoplasm of the infected cells and inhibiting the migration of PMNL across the intestinal epithelium (Bliven and Maurelli, 2012). A detailed analysis of the molecular rearrangements occurred in the *cad* operon of several EIEC strains belonging to different serotypes (Casalino et al., 2003) has shown that, similarly to *Shigella*, the silencing of the *cad* locus has been accomplished through convergent evolution. In contrast to *Shigella*, in EIEC the *cad* region is colinear with the *E. coli* K12 chromosome and the lack of cadaverine synthesis is mainly due to the inactivation of the gene encoding the CadC transcriptional regulator (Casalino et al., 2010). By comparing the *cad* loci of EIEC and *Shigella*, it appears that the rearrangements occurred in EIEC are less severe compared to the complete erosion of the locus observed in several *Shigella* strains (Casalino et al., 2005; Prosseda et al., 2007; **Figure 2**). Indeed, despite the antivirulence role played by cadaverine (Fernandez et al., 2001), emerging O96:H19 EIEC strains still maintains an integer *cad* operon and exhibits a LDC^+^ phenotype (Michelacci et al., 2016).

As compared to the commensal *E. coli* the polyamine profile of *Shigella* is affected not only by the lack of cadaverine but also by the marked accumulation of spermidine and by the loss of *N*-acetyl spermidine, the inert form of spermidine (Di Martino et al., 2013a). The increased spermidine content depends on the loss of the spermidine acetyltransferase (SAT), the enzyme encoded by the *speG* gene and responsible for the conversion of spermidine into *N*-acetylspermidine. In *Shigella* it has been demonstrated that a higher level of spermidine increases survival within macrophages and confers bacteria a higher resistance to oxidative stress (Barbagallo et al., 2011). Similarly to how observed for the *cad* locus, also *speG* silencing is the result of convergent evolution. A comparison of the polyamine profiles of several EIEC strains with those of *Shigella* and *E. coli* K12 has revealed that in EIEC major polyamines attain levels in-between those observed in *E. coli* and *Shigella*. Indeed, as compared to commensal *E. coli*, in EIEC intracellular putrescine is significantly increased and spermidine tends to be higher. Nevertheless, in contrast to *Shigella*, *N*-acetylspermidine is still present in most EIEC strains (Campilongo et al., 2014), indicating that the loss of *speG* is an emerging trait. However, when spermidine accumulation is induced in EIEC through deletion of the *speG* gene, survival within macrophages, as well as resistance to oxidative stress are increased (Campilongo et al., 2014). This confirms that the absence of SAT activity confers to intracellular bacteria like EIEC and *Shigella* an increased capability to defy antagonistic host environment. Moreover, the analysis of the polyamine profiles has revealed that the higher level of putrescine in EIEC is determined by increased transcription of *speC*, promoted by the lack of cadaverine. The *speC* gene encodes the enzyme converting L-ornithine into putrescine. On the basis of these observations it has been suggested (Campilongo et al., 2014) that during the transition toward the pathogenic phenotype, the modification of the polyamine profile might have been triggered by the loss of cadaverine, with the double effect of favoring the invasive process and increasing the putrescine level. Since putrescine is an important intermediate in the synthesis of spermidine and, consequently, of *N*-acetylspermidine, its increase may in turn have caused higher levels of both polyamines. In this scenario the silencing of *speG*, which appears completed in *Shigella* but can be regarded as an ongoing process in EIEC, would represent the last step favoring further accumulation of spermidine and the disappearance of *N*-acetylspermidine.

Another noteworthy pathoadaptive mutation in *Shigella* is the requirement for exogenous nicotinic acid (NAD) due to inactivation of the *nad* genes (Prunier et al., 2007), required for *de novo* synthesis of NAD. Also in this case the inability to synthesize NAD is not a generalized feature among EIEC strains (Di Martino et al., 2013b). In those EIEC strains requiring NAD it has been shown that the preferential target in the pathoadaptation process is the *nadB* gene, inactivated through diverse strategies, involving point mutations or IS insertions.

Altogether, the picture emerging from the observations on pathoadaptive mutations suggests that EIEC might represent intermediates in the evolution toward a full-blown phenotype, with some mutational events still confined to *Shigella* (**Figure 2**). However, a recent whole-genome comparative analysis (Pettengill et al., 2016), performed on a large number of *Shigella* and EIEC genomes, indicates that *Shigella* and EIEC evolved independently. Nevertheless, the same authors proposed that, while EIEC as a group cannot be considered the ancestor to *Shigella*, some EIEC lineages might have been the *Shigella* ancestor.

## Emerging Enteroinvasive *Escherichia coli*

The recent outbreaks occurred in Europe caused by the EIEC O96:H19 led the scientific community to reconsider the role of EIEC infection in industrialized countries (Escher et al., 2014; Michelacci et al., 2016; Newitt et al., 2016). Such EIEC serotype had never been reported before 2012 and represents a new virulent emergent clone. The EIEC O96:H19 isolated from two outbreaks occurred in Italy and United Kingdom and from a sporadic case of disease reported in Spain were studied by whole genome sequencing (Pettengill et al., 2015; Michelacci et al., 2016). The genomic analysis confirmed that all the isolates belonged not only to the same unprecedented EIEC serotype, but also to the same sequence type (ST-99), never observed before in EIEC strains (Michelacci et al., 2016). The analysis of the distribution of virulence genes typical of EIEC and *Shigella* highlighted the presence in the three strains of the plasmid genes encoding the T3SS system and its effectors, as well as the master transcriptional regulators genes *virF* and *virB*. As for the chromosomally located virulence genes, the three isolates showed the presence of the genetic determinants of a T2SS and were all negative for those encoding the aerobactin system involved in iron uptake. Interestingly, none of the O96:H19 isolates was found to have undergone the process of pathoadaptation through accumulation of the mutations described in the literature for EIEC and *Shigella* (Bliven and Maurelli, 2012; Prosseda et al., 2012). Nevertheless, the three isolates were shown to display minor differences. The plasmid profiles obtained through the genomic analysis highlighted the presence of five plasmids in the strains isolated in Spain and United Kingdom and three plasmids in that responsible of the Italian outbreak, with three plasmids in common in the three strains. Altogether, these observations strengthen the hypothesis of the emergence of a new virulent EIEC clone circulating in Europe.

Phenotypic analysis also highlighted peculiar properties of this EIEC clone, when compared to reference EIEC and *Shigella* strains. Biochemical characterization showed that the isolates displayed the LDC activity, confirming the lack of the related pathoadaptive mutations observed through genome analysis, and interestingly showed that the isolates retained the ability to ferment lactose (Michelacci et al., 2016), usually lacking in *Shigella* and in the majority of EIEC strains (Tozzoli and Scheutz, 2014). Generally, a better fitness was observed for the O96:H19 strains when comparing the growth curves with those of *Shigella* and reference EIEC strains (Michelacci et al., 2016). Moreover, swimming motility was observed for the strains from Italian and Spanish cases, which was instead completely absent in the strain from United Kingdom and in all the other EIEC and *Shigella* strains tested. Such phenotypic traits are not typical of intracellular pathogens such as EIEC and *Shigella*, while they are more common in *E. coli* strains, contributing to their great ability in surviving and adapting in different ecological niches.

These findings support the hypothesis of the evolution of EIEC and *Shigella* after the acquisition of the pINV by multiple lineages of commensal *E. coli*, followed by a multi-step adaptation process. Such an evolutionary pathway could be exemplified by EIEC ST-280 Clonal Complex, which could have been generated with the acquisition of the pINV plasmid by a commensal *E. coli* eventually evolving toward *Shigella* belonging to related clonal complexes (ST-149, 152, 243, 245, 250) (Wirth et al., 2006; Michelacci et al., 2016). The mechanism could have involved multiple events of pathoadaptive mutations, giving origin to the existing *Shigella* clones, specialized for intracellular survival with detriment of the ability to persist outside the host. A similar paradigm could also explain the emergence of other EIEC clones following the acquisition of pINV by other commensal *E. coli*. This event in some cases could be followed by the accumulation of pathoadaptive mutation, as it is the case of the EIEC strains belonging to ST-6 clonal complex, while some other clones could have maintained all the functions granting an efficient extracellular persistence, such as the EIEC O96:H19 belonging to ST-99 (Michelacci et al., 2016). The observed better fitness of EIEC O96:H19 in comparison with that of the other reference EIEC and *Shigella* strains could have favored its survival in the extracellular environment and allowed its overgrowth in the food vehicles, granting it a high potential as a foodborne pathogen, as demonstrated in the two large episodes occurred in Italy and United Kingdom (Michelacci et al., 2016; Newitt et al., 2016).

## Conclusion and Perspectives

Genomics approaches in combination with phenotypic analyses have a strong potential toward the formulation of new intriguing hypotheses on the ongoing evolution of EIEC. Currently available comparisons between EIEC and *Shigella* genomes support the need for a taxonomical revision moving the *Shigella* genus back within the *E. coli* species (Michelacci et al., 2016; Pettengill et al., 2016). As a matter of fact, *Shigella* clades are interspersed in clusters of *E. coli* genomes regardless of the bioinformatics approach used for the phylogenetic analysis (Sahl et al., 2015; Pettengill et al., 2016). In the light of recent studies, the organization of the EIEC genome appears to have been originated from multiple independent events (Hazen et al., 2016; Pettengill et al., 2016). This hypothesis finds even stronger evidence in the emergence of a novel EIEC clone belonging to O96:H19 serotype, which exhibits phenotypic traits more typical of *E. coli* than of reference EIEC or *Shigella* (Michelacci et al., 2016).

The acquisition of the plasmid may represent the first step in the emergence of new EIEC clones, but it is well known to be not sufficient for establishing the full pathogenicity (Sansonetti et al., 1983). In this context, it is of great interest to deeper investigate on the role and relevance of functions that *Shigella* has lost in its route toward an intracellular life-style but that are still retained by most EIEC strains.

## Author Contributions

BC, VM, SM, and GP proposed the idea of the review; MP, VM, MG, and RT wrote the review draft; MP, MM, and GP design the figures; BC, MM, GP, MG, and SM wrote the final version of the review. The final text has been read and approved by all the authors of the review.

## Conflict of Interest Statement

The authors declare that the research was conducted in the absence of any commercial or financial relationships that could be construed as a potential conflict of interest.

## Abbreviations

DAMP: damage-associated molecular pattern
DEC: diarrheagenic *E. coli*
HGT: horizontal gene transfer
H-NS: heat-stable nucleoid-structuring protein
IL: interleukin
IS: insertion sequence
MAPK: mitogen-activated protein kinase
NLR: Nod-like receptor
PAI: pathogenicity island
PAMP: pathogen-associated molecular pattern
pINV: virulence plasmid
PMNL: polymorphonuclear leukocytes
PRR: pattern recognition receptor
SHI: *Shigella* pathogenicity island
SRL: *Shigella* resistance locus
sRNA: small RNA
T2SS: type II secretion system
T3SS: type III secretion system
TLR: Toll-like receptor
TNF: tumor necrosis factor.
